# Medication errors involving anticoagulants: Data from the Danish patient safety database

**DOI:** 10.1002/prp2.307

**Published:** 2017-04-03

**Authors:** Jakob Nørgaard Henriksen, Lars Peter Nielsen, Annemarie Hellebek, Birgitte Klindt Poulsen

**Affiliations:** ^1^ Department of Clinical PharmacologyAarhus University HospitalAarhusDenmark; ^2^ Unit for Quality and Patient SafetyCapital Region of DenmarkCopenhagenDenmark

**Keywords:** Adverse incidents, anticoagulant therapy, clinical pharmacology, Danish patient safety database

## Abstract

Reporting of adverse incidents is mandatory in Denmark. All reported adverse incidents are made anonymously, and stored in an encrypted database. It is the purpose of this descriptive study to describe the severity of adverse medication incidents caused by oral anticoagulants in hospitals. All moderate, severe and fatal reports concerning non‐vitamin K antagonist oral anticoagulants were analyzed from date of marketing until July 8 2014. The data collection for warfarin was from January 1 2014 until July 8 2014. Three independent specialists in clinical pharmacology evaluated the severity of incident outcomes. A total of 147 adverse medication incidents were analyzed, and showed that de facto or potentially fatal and serious incidents were most frequently associated with sector change (admission to or discharge from hospital, or undergoing surgery) and resulted from insufficient or excess dosing. Physicians should be aware when prescribing and changing anticoagulant therapy to avoid severe or fatal incidents.

**Abbreviations**
DPSD: Danish Patient Safety Database
INR: international normalized ratio
NOAC: non‐vitamin K antagonist oral anticoagulant
TTR: therapeutic range

## Introduction

Anticoagulant drugs are widely used for both treatment and prophylaxis of thrombotic diseases (Adelborg et al. 2016), and are some of the drugs that most frequently cause medication errors, as demonstrated by previous data (Runciman et al. 2003; Hardmeier et al. 2004). These drugs often cause serious or fatal events (Runciman et al. 2003; Hardmeier et al. 2004; Saedder et al. 2014), and are therefore considered to be high risk medication (Danish Medicines Agency 2011). In particular, when patients are transferred from one sector to the other, unintended incidents related to medication can happen (Bell et al. 2011).

Warfarin was one of the first anticoagulant drugs on the market, and has been available since 1964 (Mercury Pharma Group Ltd 2016). In 2014, a total of 88.158 patients were treated with warfarin in Denmark (Sundhedsdatastyrelsen 2016). In March 2008, the first non‐vitamin K antagonist oral anticoagulant (NOAC), Pradaxa^®^ (dabigatran), was released (Boehringer Ingelheim 2016). Three more NOACs have since been introduced to the market; Xarelto^®^ (rivaroxaban) in September 2008, Eliquis^®^ (apixaban) in May 2011 and Lixiana^®^ (edoxoban) in June 2015 (Bayer Pharma AG 2015; Bristol‐Myers Squibb/Pfizer EEIG 2016; Daiichi Sankyo Europe BmgH 2016). As of 2014 in Denmark, a total of 23.466 patients were treated with dabigatran, 16.085 patients with rivaroxaban and 8.024 patients with apixaban (Sundhedsdatastyrelsen 2016).

The Danish Patient Safety Database (DPSD) is a national database containing all reported adverse incidents in Danish Health Care since 2004, when it was made mandatory for all health care personnel in the hospital sector to report adverse incidents (Ministry of Health/Jens Kristian Gøtrik 2003). The definition of an adverse incident is an incident or error that is unassociated to the patient's disease, but could have or, in fact, has caused harm to the patient (Government Agency of Patient Safety 2016). The reported adverse incidents are made anonymously and filed in an encrypted database, the DPSD. The purpose of this database is to generate information and provide learning objectives, thereby identifying ways to improve medication safety. Strict policy regarding the anonymity of the person making the report is formulated (Government Agency of Patient Safety 2016), and access to the information in the database is restricted.

We chose anticoagulant drugs because of an increasing use and the association with serious adverse medication incidents. Unlike earlier reports (Zaidenstein et al. 2002; Fanikos et al. 2004; Piazza et al. 2011), our data material contained NOACs as well as warfarin. It is the purpose of this study to describe the severity rather than the extent of adverse medication incidents and the circumstances that led to them, in order to provide learning objectives.

## Materials and Methods

### Design

Descriptive study.

### Setting and data source

Reports to the Patient Safety Database are free‐text fields with no minimum amount of information required to complete the reporting process.

Access to the database was obtained on July 8 2014. On this date, one abstracter manually retrieved all adverse incidents reported as moderate, serious or fatal from the hospital sector concerning dabigatran, rivaroxaban and apixaban, and manually transcribed them to a Microsoft Excel^®^‐file in anonymous form. The marketing date of these three drugs therefore became the first possible entry point into the database. For warfarin, all reported moderate, serious and fatal adverse incidents from January 1 2014 until July 8 2014 were collected. As warfarin has been marketed for several years and by far is the most commonly used anticoagulant in Denmark, adverse incidents reporting was assumed to be stable, and therefore no reports were included before January 2014. We did, however, add fatal cases concerning warfarin prior to 2014 not to miss valuable information. This added though only two more reports. Reports on incidents occurring in primary care were excluded. In some reports more than one anticoagulant was reported.

### Classification of adverse medication incidents

We sorted available information for every adverse incident into categories consisting of gender (male, female or unknown), age (0–50, 50–75, >75 or unknown), reported drug (warfarin, dabigatran, rivaroxaban, apixaban or unknown), medication process (prescribing, administering, dispensing or unknown), type of problem (excess dose, insufficient dose, other^1^ or unknown), hospitalization process (admission, in hospital, discharge, surgery, unknown) and specific clinical situation (bridging, medication review, monitoring and other; see Table 2 for definitions).

This supported analysis of the involved drug, the specific process of medication and the situation during hospitalization, where the adverse incidents were most harmful. Incidents that did not fit into a category were labeled unknown.

### Analysis

Every adverse incident was graded according to severity by three independent specialists in Clinical Pharmacology. They graded incidents with both known and unknown outcomes according to the definitions in Table 1.

**Table 1:** Definitions of severity of the adverse incidents

Category	Known outcome (Government Agency of Patient Safety 2016)	Unknown outcome (Lisby et al. 2005)
No harm/mild/potentially nonsignificant	No harm/minor harm that does not require additional treatment or care.	Medication error that is judged without clinical risk for the patient.
Moderate/potentially significant	Transient harm that requires admission to hospital, treatment by a physician, increased level of care or additional treatment for hospitalized patients.	Medication error that is judged to pose a potential clinical risk of being inconvenient to the patient, without permanently harming the patient.
Serious/potentially serious	Permanent harm that requires admission to hospital, treatment by a physician, increased level of care or additional treatment for hospitalized patients. This includes harms that require acute, life‐saving treatment.	Medication error that is judged to be able to cause a potential clinical risk of harming the patient.
Fatal/potentially fatal	Fatal	Medication error that is judged to pose a potential clinical risk of leading to a fatal outcome.

Adverse medication incidents with a known outcome (an actual harm to the patient) was described as a “de facto” harm, whereas adverse medication incidents with an unknown outcome (and a potential for harming the patient) was described as a potential harm. If grading differed among the clinical pharmacologists, the reported adverse incident was reviewed again by the group to achieve consensus. We chose to pool both de facto and potentially nonsignificant, significant, serious or fatal to increase the strength of the data.

Thus, an adverse incident with a known outcome described in the database as “A 48‐year old woman with known systemic lupus erythematosus and antiphospholipid antibody syndrome had previously been switched from warfarin to dabigatran due to compliance problems. She was admitted due to bleeding and dabigatran was discontinued. This was not reinstated upon discharge. Seven days later, the patient was admitted to hospital with pulmonary embolism” would be categorized as follows: (1) Age = 48; (2) gender = female; (3) reported drug = dabigatran; (4) medication process = prescribing; (5) type of problem = no anticoagulant therapy; (6) location in the health care sector = discharge; (7) specific clinical situation = medication review; (8) outcome = 7 days later admitted with a lung embolus. This case was judged by the clinical pharmacologists as serious.

An adverse incident with an *unknown* outcome described in the database as “An 88‐year old woman was admitted with suspected deep vein thrombosis. The ultrasound was negative. She was discharged with both dabigatran and low molecular weight heparin” would be categorized as follows: (1) Age = 88; (2) gender = female; (3) reported drug = dabigatran; (4) medication process = prescribing; (5) type of problem = excess anticoagulant therapy; (6) location in the health care sector = discharge; (7) specific clinical situation = medication review; (8) outcome = *unknown*. This case was judged by the clinical pharmacologists to be potentially serious.

## Results

**Figure 1:**
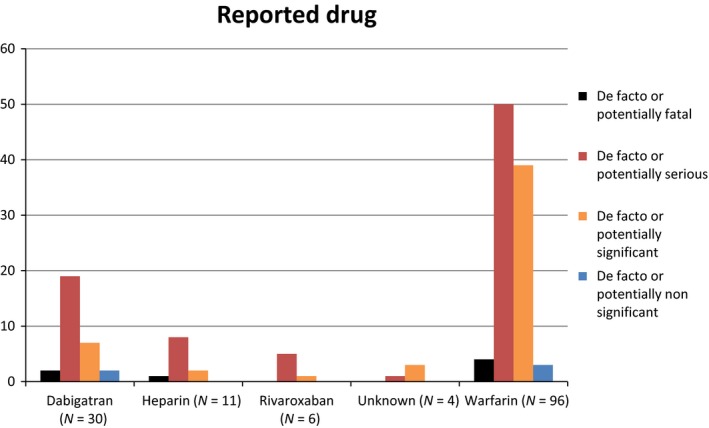
Reported drug and associated severity. Direct comparisons between individual drugs should be avoided, as the basis for data could be different.

A total of 148 adverse incidents were included in the study. One was omitted due to lack of information, and was therefore not possible to grade. Of the remaining 147, the outcome was known in 65 incidents (44%) and unknown in 82 (56%). In total, there were (de facto or potentially) seven fatal, 83 serious, 52 significant and five nonsignificant adverse medication incidents (Fig. 1).

In the medication process, an adverse medication incident occurred during prescription in 115 of the total 147 incidents (78%). All the seven fatal adverse medication incidents occurred in this part of the medication process and also accounted for 70 of the total 83 serious adverse medication incidents (84%). Vice versa, there were no fatal incidents due to administration and dispensation, and only one and six, respectively, were graded as serious.

Excess or insufficient dosing was associated with all the seven fatal adverse medication incidents in our data material, with most being caused by excess doses (5 of 7). In the subcategories of excess or insufficient dosing, 33 (40%) and 23 (28%), respectively, of a total of 83 were graded as de facto or potentially serious.

In the hospitalization process, described as sector change (admission, discharge and surgery) was associated with a total of 116 of 147 adverse medication incidents (79%) as well as all the fatal (100%) and 68 out of the total 83 serious (82%) adverse medication incidents.

Important parts of the physician's task when handling patients during sector change (bridging, monitoring and medication review) was associated with all seven fatal (100%) and 70 of 83 serious adverse medication incidents (84%). Two of these 70 serious adverse medication incidents were thus not related to sector change (3%).

Compared to admission and surgery, the discharge phase of the hospitalization process was associated with more adverse medication incidents (63 of 147 = 43%) than the two others (22 and 31 of 147 respectively = 15 and 21%). Only three of the 147 adverse medication incidents (2%) in the category “hospitalisation process” did not contain sufficient information to fit into a subcategory. It therefore became the most reliable category in terms of amount of data therein.

**Figure 2:**
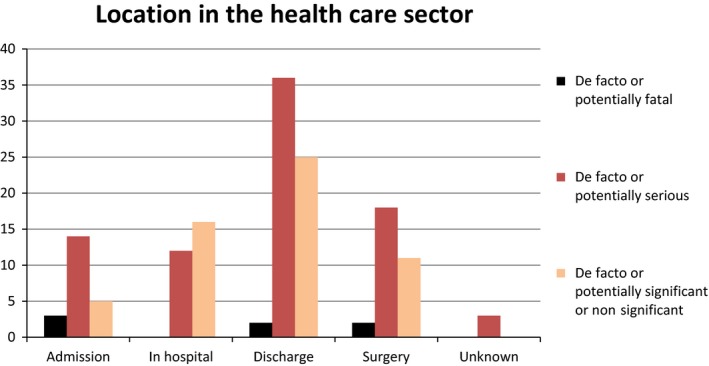
The situation in which the adverse incident happened related to severity of outcome.

Where information in the reports about dosing errors was available, the adverse medication incident in the admission phase was due to excess anticoagulant in seven of 11 incidents (64%) and insufficient anticoagulant in the remaining four (36%). During surgery, the adverse medication incident was due to excess anticoagulant in 17 of the 28 incidents (61%) with insufficient anticoagulant accounting for the remaining 11 (39%). During discharge, the numbers were 19 of 43 (44%) and 24 of 43 (56%), respectively (results not shown Fig. 2).

## Discussion

Our data showed that all fatal and almost all serious adverse medication incidents were associated with the prescribing phase of the medication process. Unlike an American study looking at reported adverse incidents over a 3‐year period in the national database (Santell et al. 2003), we did not find a similar association with neither administering nor dispensing. This indicates a problem related to physicians rather than the nursing staff. It poses a question whether better decision‐making support, more education or other actions are necessary when trying to minimize adverse medication incidents. In some regions of Denmark, the electronic patient chart allows the physician to use a function called “anticoagulation” to evaluate international normalized ratio (INR) values and warfarin given while the patient is in hospital. This helps to regulate anticoagulant therapy with warfarin while the patient is hospitalized. However, this feature only includes patients treated with warfarin and no information about doses prior to admission is given.

This leads to our second major finding. Sector change (i.e., admission, discharge and surgery) was associated with the highest number of serious and fatal adverse medication incidents. This is supported by the adverse medication incidents related to prescribing situations such as bridging, critical evaluation of the patients' medication in a medication review and monitoring anticoagulant therapy. During admission and surgery, prescribing excess anticoagulant was the most frequent problem. During discharge, the reverse was true with prescribing insufficient anticoagulant being the most frequent problem.

With recent data showing no increased benefit of bridging low to moderate risk patients on warfarin, but instead an increased risk of bleeding (Douketis et al. 2015), fewer patients are expected to be offered bridging with low molecular weight heparin and thereby reducing the risk of adverse medication incidents.

We know there is an increased risk of unintended discontinuation of evidence‐based therapy in patients admitted to hospital (Bell et al. 2011), and it has also been demonstrated, that among the five categories of drugs studied, anticoagulant therapy had the highest adjusted odds ratio of unintended discontinuation (Bell et al. 2011). Likewise, it has been documented, that adverse incidents related to medication increase with a patient's admittance to hospital for any reason (Cornish et al. 2005). Work is being done to implement an electronic information system showing a patient's current medication that is accessible to physicians in both primary care and hospitals. However, it is not yet fully implemented among all general practitioners and is not yet fully equipped to handle information about varying doses of warfarin for example.

While it has been shown that anticoagulant therapy along with cardiovascular and antiasthmatic therapy were the most frequent medications to cause fatalities among hospitalized patients (Ebbesen et al. 2001), there were no fatalities among hospitalized patients in our data set.

Our data was not able to support NOACs being associated with different risk patterns compared with warfarin, and data were too limited to report on their own. This was not only due to the low number of events for NOACs, but also because of the quality of the reported incidents. In almost all the reports, we had no information about who prescribed the medication, the time in therapeutic range (TTR) for warfarin, patients' co‐morbidity and co‐medication. An attempt to do a head to head comparison between warfarin and NOACs would only be associated with a strong possibility of misinterpretation without bringing clinically relevant data. The data on NOACs merely confirm that the risk of harming patients with anticoagulants is increased in specific clinical situations such as sector change, particularly during discharge.

A major challenge in our data material proved to be the level of information contained in the reports. When reporting an adverse incident to the Patient Safety Database, a health care person is prompted to choose either a known or an anticipated degree of severity as well as to provide information regarding the location and time of the incident. The options for harm are none, mild, moderate, serious or fatal (Table 1). Although a description of events prior to the adverse incident is required, there are no requirements about the quality or extent of the description. The level of information regarding the patient and the incident therefore varies significantly between reports. Therefore, we were unable to fully characterise all adverse incidents according to predefined categories. Naturally, this inconsistency of information, tended to inflict and to some degree incapacitate the severity analysis.

Furthermore, more than half the incidents had an unknown outcome, and analysis depended on anticipation of a certain outcome rather than on a factual outcome.

Due to these limitations, we were not able to distinguish between the different classes of drugs in terms of severity or to draw any conclusions about the individual drugs and the individual characteristics about the problem (for example, which drugs were insufficiently dosed, did the patient have renal impairment or when were INR‐values insufficiently handled). Concerning warfarin, though, we know that from a combined retrospective and prospective Danish study that disease and drug‐drug interactions were the two most common reasons for patients treated with warfarin being admitted to hospital due to elevated INR‐values with or without an active bleeding (Meegaard et al. 2012).

Although reporting of serious adverse effects as well as adverse medication incidents are mandatory in Denmark, we must assume some degree of underreporting. In a randomized controlled trial of patients with atrial fibrillation for example, 2,71% and 3,36% of patients on dabigatran and warfarin, respectively experienced a major bleeding event per year as an adverse effect of their treatment (Connolly et al. 2009). However, postmarketing reporting of these adverse effects is much lower. When comparing the presumed annual major bleeding events rate in patients on warfarin using the data from the above‐mentioned trial with actual reporting to the Federal Drug Administration in the United States, only about a third of these events are reported (Moore and Bennett 2012). Therefore, the full scope of adverse medication incidents with anticoagulants might very well be larger than our data indicate.

We chose not to look at the extent but rather the severity of the adverse medication incidents, and therefore only included adverse incidents related to warfarin from January 2014 until July 2014 with the addition of two, fatal incidents before that. This created a longer period for adverse incidents to be recorded for NOACs relative to warfarin. However, warfarin was up until July 2014 still used substantially more than NOACs (Sundhedsdatastyrelsen 2016) and the relative longer time span of possible reported adverse incidents with NOACs is likely balanced by the much higher consumption of warfarin. We have attempted to highlight this in Table 2. Whether a change in the type and severity of adverse incidents has changed in the years leading up to 2014 is unknown, but since warfarin has been marketed for decades, this would not seem likely.

**Table 2:** Number of adverse incidents divided into categories, subcategories as well as severity

Categories	Total number within the subgroup (%)	De facto or potentially fatal (%)	De facto or potentially serious (%)	De facto or potentially significant+nonsignificant (%)
Gender				
Male	55 (37%)	3 (6%)	31 (56%)	21 (38%)
Female	62 (42%)	2 (3%)	40 (65%)	20 (32%)
Unknown	30 (21%)	2 (7%)	12 (40%)	16 (53%)
Age				
0–50	9 (6%)	0	5 (56%)	4 (44%)
51–75	43 (29%)	1 (3%)	26 (60%)	16 (37%)
75+	60 (41%)	4 (7%)	37 (62%)	19 (31%)
Unknown	35 (24%)	2 (6%)	16 (46%)	17 (48%)
Reported drug				
Warfarin	96 (65%)	4 (4%)	50 (52%)	42 (44%)
Dabigatran	30 (21%)	2 (7%)	19 (63%)	9 (30%)
Heparin	11 (7%)	1 (9%)	8 (73%)	2 (18%)
Rivaroxaban	6 (4%)	0	5 (83%)	1 (17%)
Unknown	4 (3%)	0	1 (25%)	3 (75%)
Medication process				
Prescribing	115 (78%)	7 (6%)	70 (61%)	38 (33%)
Administering	4 (3%)	0	1 (25%)	3 (75%)
Dispensing	16 (11%)	0	6 (38%)	10 (62%)
Unknown	12 (8%)	0	6 (50%)	6 (50%)
Type of problem				
Excess anticoagulant	51 (35%)	5 (10%)	33 (65%)	13 (25%)
Insufficient anticoagulant	50 (34%)	2 (4%)	23 (46%)	25 (50%)
Other^a^	9 (6%)	0	6 (67%)	3 (33%)
Unknown	37 (25%)	0	21 (57%)	16 (43%)
Hospitalization process				
Admission	22 (15%)	3 (14%)	14 (64%)	5 (22%)
In hospital	28 (19%)	0	12 (43%)	16 (57%)
Discharge	63 (43%)	2 (3%)	36 (57%)	25 (40%)
Surgery	31 (21%)	2 (6%)	18 (58%)	11 (36%)
Unknown	3 (2%)	0	3 (100%)	0
Specific clinical situation				
Bridging^b^	25 (17%)	2 (8%)	16 (64%)	7 (28%)
Medication review^c^	61 (41%)	2 (3%)	36 (59%)	23 (38%)
Monitoring^d^	22 (15%)	3 (14%)	18 (82%)	1 (4%)
Other^e^	39 (27%)	0	13 (33%)	26 (67%)

The numerals state the number of adverse incidents in the listed category. Percentages in the second column from the left are percent within the category. Percentages in the third, fourth and fifth column from the left are percentages in the subcategory.

a I.e. Patient given one anticoagulant, but should have been given another or not giving antidote to warfarin with a high INR.

b Bridging was defined as changing anticoagulant therapy for a short while (i.e. during surgery) from one anticoagulant to another.

c Medication review consisted of: Physician's review and prescribing of patient's medicine at admission, during hospital stay or discharge.

d Monitoring was defined as reacting to an international normalized ratio (INR)‐value.

e Missing or incorrect information in the electronic patient chart, lack of compliance or dispensing error.

Instead of highlighting differences between the drugs, our data showed anticoagulants as a whole to be associated with serious and fatal harm to patients during sector change, particularly during discharge. Our data showed that the fatal and serious problems were with prescribing rather than administering and dispensing the drugs.

With the purpose of providing learning points to the health care system, the Danish Patient Safety Database should support extra careful attention when prescribing anticoagulants and caring for patients treated with them during admission, discharge and surgery.

## Conclusion

Adverse incidents on anticoagulant therapy are associated with serious and even fatal harm to patients. In our dataset, we identified two main areas that caused serious and fatal outcomes. First of all, the prescription phase of the medication process proved to be the clinically most important in causing harm. We did not find similar relationship to administering and dispensing phases. Secondly, sector transfer, that is, admission, discharge and surgery, was associated with the majority of fatal and serious incidents. Our data do not support that the introduction of NOACs has influenced this significantly.

In all, anticoagulants have to be considered as risk medication in general, and patients treated with such drugs warrant increased attention.

## Disclosure

Dr. Henriksen reports nonfinancial support from LEO Pharma, outside the submitted work.
